# Expression of virulence and antimicrobial related proteins in *Burkholderia mallei* and *Burkholderia pseudomallei*

**DOI:** 10.1371/journal.pntd.0011006

**Published:** 2023-01-06

**Authors:** Armand Paauw, Holger C. Scholz, Roos H. Mars-Groenendijk, Lennard J. M. Dekker, Theo M. Luider, Hans C. van Leeuwen

**Affiliations:** 1 Netherlands Organization for Applied Scientific Research TNO, Department of CBRN Protection, Rijswijk, The Netherlands; 2 Centre for Biological Threats and Special Pathogens, Highly Pathogenic Microorganisms (ZBS 2), Robert Koch Institute, Berlin, Germany; 3 Department of Neurology, Erasmus MC, Rotterdam, The Netherlands; Colorado State University, UNITED STATES

## Abstract

**Background:**

*Burkholderia mallei* and *Burkholderia pseudomallei* are both potential biological threat agents. Melioidosis caused by *B*. *pseudomallei* is endemic in Southeast Asia and Northern Australia, while glanders caused by *B*. *mallei* infections are rare. Here we studied the proteomes of different *B*. *mallei* and *B*. *pseudomallei* isolates to determine species specific characteristics.

**Methods:**

The expressed proteins of 5 *B*. *mallei* and 6 *B*. *pseudomallei* strains were characterized using liquid chromatography high-resolution tandem mass spectrometry (LC-HRMS/MS). Subsequently, expression of potential resistance and virulence related characteristics were analyzed and compared.

**Results:**

Proteome analysis can be used for the identification of *B*. *mallei* and *B*. *pseudomallei*. Both species were identified based on >60 discriminative peptides. Expression of proteins potentially involved in antimicrobial resistance, AmrAB–OprA, BpeAB–OprB, BpeEF–OprC, PenA as well as several other efflux pump related proteins and putative β-lactamases was demonstrated. Despite, the fact that efflux pump BpeAB–OprB was expressed in all isolates, no clear correlation with an antimicrobial phenotype and the efflux-pump could be established. Also consistent with the phenotypes, no amino acid mutations in PenA known to result in β-lactam resistance could be identified.

In all studied isolates, the expression of virulence (related) factors Capsule-1 and T2SS was demonstrated. The expression of T6SS-1 was demonstrated in all 6 *B*. *pseudomallei* isolates and in 2 of the 5 *B*. *mallei* isolates. In all, except one *B*. *pseudomallei* isolate, poly-beta-1,6 N-acetyl-D-glucosamine export porin (Pga), important for biofilm formation, was detected, which were absent in the proteomes of *B*. *mallei*.

Siderophores, iron binding proteins, malleobactin and malleilactone are possibly expressed in both species under standard laboratory growth conditions. Expression of multiple proteins from both the malleobactin and malleilactone polyketide synthase (PKS) and non-ribosomal peptide synthetase (NRPS) clusters was demonstrated in both species. All *B*. *pseudomallei* expressed at least seven of the nine proteins of the bactobolin synthase cluster (bactobolin, is a ribosome targeting antibiotic), while only in one *B*. *mallei* isolate expression of two proteins of this synthase cluster was identified.

**Conclusions:**

Analyzing the expressed proteomes revealed differences between *B*. *mallei* and *B*. *pseudomallei* but also between isolates from the same species. Proteome analysis can be used not only to identify *B*. *mallei* and *B*. *pseudomallei* but also to characterize the presence of important factors that putatively contribute to the pathogenesis of *B*. *mallei* and *B*. *pseudomallei*.

## Author summary

Meliodosis and glanders are both potential biological threat agents. Glanders is endemic in Southeast Asia and Northern Australia but occasionally found in other countries. Meliodosis is caused by *Burkholderia pseudomallei*, while glanders is caused by *Burkholderia mallei*. The diagnosis of these diseases is difficult because the clinical picture varies and the causative agents are difficult to identify. There is no vaccine available and antimicrobial therapy is lengthy and with an uncertain outcome.

This study was conducted to determine whether certain characteristics are or aren’t expressed by multiple isolates from the same species. Therefore, the proteins expressed by 5 *B*. *mallei* and 6 *B*. *pseudomallei* were characterized using LC-HRMS/MS. Results of this study demonstrated;

- The homogeneity in *B*. *mallei* isolates and the diversity in *B*. *pseudomallei* isolates.
- That proteomic analysis can be used to identify *B*. *pseudomallei* and *B*. *mallei*.
- The need to study multiple isolates of a pathogen to determine whether a particular virulence factor or antimicrobial related protein is important for the species to be pathogenic or confer antimicrobial resistance.
- The expression of multiple virulence factors, proteins of several PKS/NRPS clusters and antimicrobial related proteins was demonstrated. No relation between phenotype and antimicrobial proteins could be made.

The demonstrated expression of virulence factors in all isolates can be used for the development of new diagnostic assays or drug development. Proteome analysis of infectious bacteria can be used to identify the species but also to characterize the presence of important factors that putatively contribute to the pathogenesis of the species.

## Introduction

In 1882 *Burkholderia mallei* was discovered as the causative agent of glanders by Loeffler and Schütz [1]. It primarily afflicts horses, mules and donkeys but can also be transmitted to humans by direct contact with infected animals or via aerosols. Because horses and donkeys are no longer involved in everyday life, glanders is rare in humans these days. Intriguingly, *B*. *mallei* is a clonal phylogenetic clade within the highly genetic diverse species *Burkholderia pseudomallei*. In contrast to *B*. *mallei*, *B*. *pseudomallei* is a saprophyte that can be isolated from soil, mud and surface water in endemic regions. Infections with *B*. *pseudomallei* lead to melioidosis, which is often severe and fatal when untreated [2, 3] and even with prolonged antimicrobial treatment-regimens around 10% fails [4]. *B*. *pseudomallei* was first isolated and also described as a “glanders-like” disease by Whitmore in 1911 [5, 6]. Most cases of melioidosis originate in Southeast Asia, where it is a common cause of pneumonia, and Northern Australia [7]. However, occasionally indigenous cases of melioidosis are also diagnosed in the Americas and Africa [8–11]. In addition, *B*. *pseudomallei* infections are detected in travelers returning from the tropics [12]. Melioidosis is a neglected tropical disease, which may spread, creating the risk that this disease will become endemic in more parts of the world. Furthermore, it is considered a potential biological threat for which no vaccine is available [2, 13].

Diagnosis can be cumbersome because the clinical manifestations of the disease varies between patients, isolation of the bacterium can be complicated in some clinical specimens and trustworthy identification to the species level is sometimes challenging [13, 14]. With proteome analysis, *B*. *mallei* and *B*. *pseudomallei* can be identified quickly and with certainty [15].

The whole or partial fractions (secretome, outer membrane proteome) of expressed proteomes of *B*. *mallei* and *B*. *pseudomallei* have been studied [3, 16–20]. However, these studies have been conducted on one strain or derivates of the particular strain studied. Since, the genetic diversity between *B*. *pseudomallei* isolates is high, it could be expected that there is also a large variation between the expressed proteomes of these isolates. Determining whether certain characteristics are expressed by multiple isolates indicate whether the expression can be used for characterization of a species or strain. Moreover, characteristics that are expressed by all isolates of a particular species might be interesting targets for detection assays or therapy development. Furthermore, studying the expression in multiple isolates of one species helps to determine whether a characteristic contributes to the virulence of that species. For example, if a virulence factor is only expressed by one isolate it isn’t likely essential for a pathogenic species to cause infection. The main objective therefore was to determine if virulence, antimicrobial resistance, polyketide and non-ribosomal peptide synthetase (PKS/NRPS) related proteins are expressed by all or a subset of the *B*. *mallei* and *B*. *pseudomallei* isolates. Therefore, the proteins expressed by 5 *B*. *mallei* and 6 *B*. *pseudomallei* were characterized using LC-HRMS/MS. Subsequently, expression of potential resistance and virulence related characteristics were analyzed and compared.

## Material and methods

### Strains

*B*. *mallei* and *B*. *pseudomallei* strains used in the study are described in Table 1. Except strain BM649, which was obtained via NCTC, all strains were obtained via the EDA project “Database B” (No. B-0060-ESM4-GC). Species identification was confirmed by MALDI-TOF MS and LC-HRMS/MS (proteome2pathogen.com) [15].

**Table 1:** Strains included in the study.

TNO-number	Species	Aliases	Collection year	Country of isolation	Host	MLST-ST
BM1343	*B*. *mallei*	L3_0762, NCTC 3709, 106	1932	India	Horse	40
BM1345	*B*. *mallei*	L3_2399, UAE 7, 6SK2, Al Ain Dubai	2004	United Arab Emirates	unknown	40
BM1347	*B*. *mallei*	L3_0586, Zagreb	1996	Yugoslavia	unknown	40
BM1349	*B*. *mallei*	L3_0558, ATCC 23344T	1944	China	human	40
BM1352	*B*. *mallei*	L3_0764, NCTC_120 (Lister strain)	1920	United Kingdom	unknown	40
BM1354	*B*. *pseudomallei*	Holland	unknown	unknown	unknown	96
BM1355	*B*. *pseudomallei*	PITT 521	1988	Pakistan	Human	72
BM1357	*B*. *pseudomallei*	06-711	unknown	unknown	unknown	1888
BM1360	*B*. *pseudomallei*	PITT 5691	unknown	unknown	unknown	6
BM1361	*B*. *pseudomallei*	006-00772/2003	unknown	unknown	unknown	96
BM649	*B*. *pseudomallei*	NCTC 04845	1935	Singapore	Monkey	51

### Genotyping of isolates with multilocus sequence typing (MLST) analysis

MLST analysis was performed according to Godoy *et al*. [21]. Internal fragments of *ace*, *gltB*, *gmhD*, *lepA*, *lipA*, *narK* and *ndg* were PCR amplified and sequenced. Each sequence variant of a locus was assigned an allele number by matching the sequences with the PubMLST *Burkholderia pseudomallei* database [22]. Next, MLST profiles were retrieved of strains commonly used in the field (K96243, 1026b and ATCC 23344 and the profiles of all 8 MLST sequence types with >100 records in the PubMSLT db [22].

The allele profiles were entered into BioNumerics version 6.6 software (Applied-Maths, Belgium) as character values. Genetic relationship between isolates was constructed using categorical clustering (values) and clustered with unweighted pair group method with arithmetic mean (UPGMA) using default settings.

### Antimicrobial susceptibility testing (E-test)

Minimum inhibitory concentrations (MIC) was performed by E-test (bioMérieux, Marcy-L’Etoile, France) following the manufacturer’s instructions. An 0.5 McFarland suspension was made in 10 mL Dulbecco’s phosphate-buffered saline (DPBS, Lonza, Walkersville, USA). This method of testing has previously shown to be comparable with the gold standard of microbroth dilution [23–26]. MICs were read after 48 h of incubation in air at 35°C.

Interpretation of susceptible, intermediate and resistance was based on Clinical and Laboratory Standards Institute (CLSI) approved guidelines for amoxicillin-clavulanate (≤8/4 S,16/8 I and ≥32/16 R), ceftazidime (≤8 S, 16 I and ≥32 R), imipenem, doxycycline(≤4 S, 8 I and ≥16 R) and trimethoprim-sulfamethoxazole (≤2/38 S, ≥4/76 R) [27]. By lack of an official MICs breakpoint for *B*. *pseudomallei* for ciprofloxacin, piperacillin, meropenem and chloramphenicol the MIC breakpoints for other non-Enterobacteriales were used as a reference. For ciprofloxacin (≤1 S, 2 I and ≥4 R), piperacillin (≤16 S, 32–64 I and ≥128 R), meropenem (≤4 S, 8 I and ≥16 R) and chloramphenicol (≤8 S, 16 I and ≥32 R). For ampicillin/sulbactam the MIC breakpoints for *Acinetobacter* sp. and *Enterobacteriaceae* was used ((≤8/4 S, 16/8 I and ≥32/16 R) [25, 28]. For tigecycline US FDA approved breakpoints for *Enterobacteriaceae* was used (≤2 S, 4 I and ≥8 R) [25].

### Sample preparation for LC-HRMS/MS analysis

Sample preparation was executed using a modified filter aided sample preparation (FASP) protocol as described, previously, all in one experiment [15, 29]. The only difference was that bacteria were harvested from a blood agar culture plate in contrast to the blood culture flasks. After the blood agar culture plate was incubated overnight at 35°C, a small sample of the culture material was resuspended in 300 μL lysis buffer (4% SDS, 100 mM DTT, 100 mM Tris-HCl, pH 8). Sample cleanup was executed using ZipTip (Merck Millipore, Darmstadt, Germany). The sample was eluted twice with 10 μL 50% acetonitrile/0.1% TFA in water in a new low binding tube (final volume 20 μL). Finally, the liquid was evaporated with the SpeedVac (Thermo Fisher Scientific, Munich, Germany). The resulting pellet was resuspended in 50 μl 1% TFA for LC-HRMS/MS analysis.

### LC-HRMS/MS analysis

LC-HRMS/MS measurements were performed on an RSLC nano-LC system (Thermo Fisher Scientific) online coupled to an Orbitrap Fusion Lumos mass spectrometer (Thermo Fisher Scientific). Samples were loaded on a C18 trap column (C18 PepMap, 300 μm ID ×5 mm, 5 μm particle size, 100 Å pore size; Thermo Fisher Scientific), and desalted for 8 min using a flow rate of 20 μL 0.1% TFA (in water)/min. The trap column was switched online with the analytical column (C18 PepMap, 75 μm ID × 250 mm, 1.8 μm particle and 100 Å pore size; Thermo Fisher Scientific) and peptides were eluted with the following binary gradient: 4%–38% solvent B in 90 minutes, where solvent A consists of 0.1% aqueous formic acid in water and solvent B consists of 80% acetonitrile and 0.08% aqueous formic acid. Column flow rate was set to 300 nL/min and column oven temperature to 40°C. For mass spectrometry detection a data-dependent acquisition method was used: a high resolution survey scan from 375 to 1,500 Th was performed in the Orbitrap (value of target of automatic gain control AGC 4e5; resolution 120,000 at 400 m/z; lock mass was set to 445.120025 Th (protonated (Si(CH_3_)_2_O)_6_). Based on this survey scan, most intense ions were consecutively isolated and fragmented until a duty cycle duration of 3 s was reached (‘Top speed’ setting). Settings for MS/MS fragmentation were: AGC target set to 1e4 ions (50 ms maximal fill time); isolation width of 1.6 u; fragmentation by HCD applying 30% normalized collision energy; detection of fragment ions in the linear ion trap. After precursors were selected for MS/MS, they were excluded within a tolerance range of 10 ppm for further MS/MS analysis for 1 min.

The mass spectrometry proteomics data is deposited at the ProteomeXchange Consortium via the PRIDE [30, 31] partner repository with the dataset identifier PXD028126.

### Proteomic data analysis

All MS-data obtained from the Orbitrap Fusion Lumos were analyzed using the settings as described in S1 Table in PEAKS 7.5. To assess specific properties of tested *B*. *mallei and B*. *pseudomallei* isolates, the MS-data was matched against several custom databases. Only peptides with a false discovery rate (FDR) ≤ 0.1% were used for further analysis.

### Proteome for bacterial identification

To conform the identity of tested bacteria, the first analysis was executed as previously described [15]. Measured MS/MS-spectra were matched using the MS2peptides-DB in PEAKS 7.5, (settings see S1 Table). Next, from each measurement a comma separated file (.csv) containing a list of identified peptides was uploaded and analyzed employing proteome2pathogen [15].

Next, to increase the number of identified peptides derived from the tested *B*. *mallei* and *B*. *pseudomallei*, a specific database was created with all annotated proteins of *B*. *mallei* and *B*. *pseudomallei* in the SwissProt database (BPC-DB) (2018-October-17). Subsequently, measured MS/MS-spectra were matched using the BPC-DB in PEAKS 7.5.

### Analysis of resistance related proteins

To examine if the expressed proteome contain proteins that could contribute to resistance related proteins several searches were executed. For β-lactam and/or carbapenem resistance, BPC-DB proteins that contained the word “beta-lactamase” were included. To prevent that similar proteins were identified multiple times, an alignment of the identified β-lactamases or carbapenemases was executed. Next, based on the number of identified (unique) peptides it was determined which type or types of β-lactamase(s) are produced by the investigated micro-organism.

For trimethoprim-sulfamethoxazole (co-trimoxazole) expressed peptides of resistance related proteins, BPC-DB proteins corresponding to dihydropteroate synthase (DHPS) and dihydrofolate reductase were analyzed for specific resistance conferring mutations. For tigecycline related resistance related, MS-data was examined for the presence of peptides that could be related to tigecycline (chemically related to tetracycline) enzymatic inactivation proteins and ribosomal protection proteins with tetracycline conferring mutations [32].

For efflux pumps potentially involved in antibiotic resistance, BPC-DB proteins that contain the word “efflux” were identified. For each of the identified proteins, the number of unique peptides were determined. Only proteins with 2 or more peptides identified are reported.

### Analysis of expressed virulence related proteins

A database and data analysis pipeline were constructed that are able to determine whether peptides are derived from (putative) virulence related proteins. The analysis is based on the database from Jian Yang (China) VFDB (Virulence Factors DataBase), which can be accessed at http://www.mgc.ac.cn/VFs/ [33]. The VFDB contains genes that are putatively involved in virulence of different bacterial species. Next, the VFDB also contains information, mainly scientific publications, on the presumed relation of the gene (and protein) to virulence of the bacterium. The database consists of two sub-databases: Database A and Database B. Database A is the core dataset and includes genes associated with experimentally verified virulence factors only, whereas the full dataset (Database B) covers all genes related to known and predicted virulence factors. A database (BPC-virulence DB) was constructed based on Database B from VFDB. This database was used to screen which peptides of proteins are present in standard cultures of *B*. *mallei* and *B*. *pseudomallei* strains.

Virulence related proteins were identified based on two or more identified peptides using PEAKS DB search using the BPC-virulence DB. To remove redundancy from the results, per unique gene name (e.g. *wcbR*) only the result with the highest number of peptides (max) was reported. At an equal number of identified peptides, the result with the highest -10LogP value of the identified protein was reported. Subsequently, the identified proteins were classified by the virulence factor to which the protein belongs.

### Proteins involved in polyketide and non-ribosomal peptide synthetase

The DB search results generated with the BPC-virulence DB were not effective in determining which proteins involved in the production of secondary metabolites are expressed. First, a literature search was executed to find potentially expressed PKS/NRPS clusters in *B*. *mallei* and *B*. *pseudomallei* [7, 34–38]. To determine if proteins involved in PKS/NRPS clusters were expressed the number of identified peptides in proteins involved in putative PKS/NRPS clusters were determined. Table 2 describes the PKS/NRPS clusters, of which we examined whether the expression of proteins could be demonstrated. The accession numbers of the proteins of the putative PKS/NRPS were searched in exported protein lists from PEAKS. When two or more peptides of a protein were identified this protein was considered present.

**Table 2:** Analyzed PKS/NRPS clusters for putative expression based on expressed proteins.

Accession in K96243	No. of CDSs	Description of PKS/NRPS cluster	Reference
Chromosome 1			
BPSL1710–BPSL1727	18	Putative nonribosomal peptide synthase (NRPS) cluster	(7, 39)
BPSL1774–BPSL1787	14	Malleobactin synthase cluster	(38)
BPSL2214–BPSL2233	21	Putative NRPS cluster.	(39)
Chromosome 2			
BPSS0130	1	Putative polyketide synthase (PKS); putative terphenyl	(7, 39)
BPSS0160	1	putative Isonitrile	(37)
BPSS0297–BPSS0312	13	Malleilactone synthase cluster	(35, 39)
BPSS0481–BPSS0487	7	Possible PKS/NRPS cluster	(7)
BPSS0581–BPSS0588	8	Pyochelin siderophore biosynthesis cluster similar to pyochelin biosynthesis cluster in *Pseudomonas aeruginosa*	(7, 39)
BPSS1006–BPSS1011	6	Putative antibiotic PKS/NRPS.	(7, 39)
BPSS1166–BPSS1174	9	Bactobolin synthase cluster	(34, 39)
BPSS1181–BPSS1199	17	Putative antibiotic PKS/NRPS.	(39)
BPSS1266–BPSS1274	9	Glidobactin synthase cluster or Syr-Bactin synthase cluster	(36, 39)
BPSS1631–BPSS1634	4	Putative lipopeptide antibiotic NRPS cluster.	(7, 39)
BPSS1805–BPSS1815	11	Miscellaneous cluster, possibly involved in the biosynthesis of a nonproteinogenic amino acid	(39)
BPSS2324–BPSS2329	6	Possible PKS/NRPS cluster	(7, 39)

## Results

### MLST analysis

**Fig 1:**
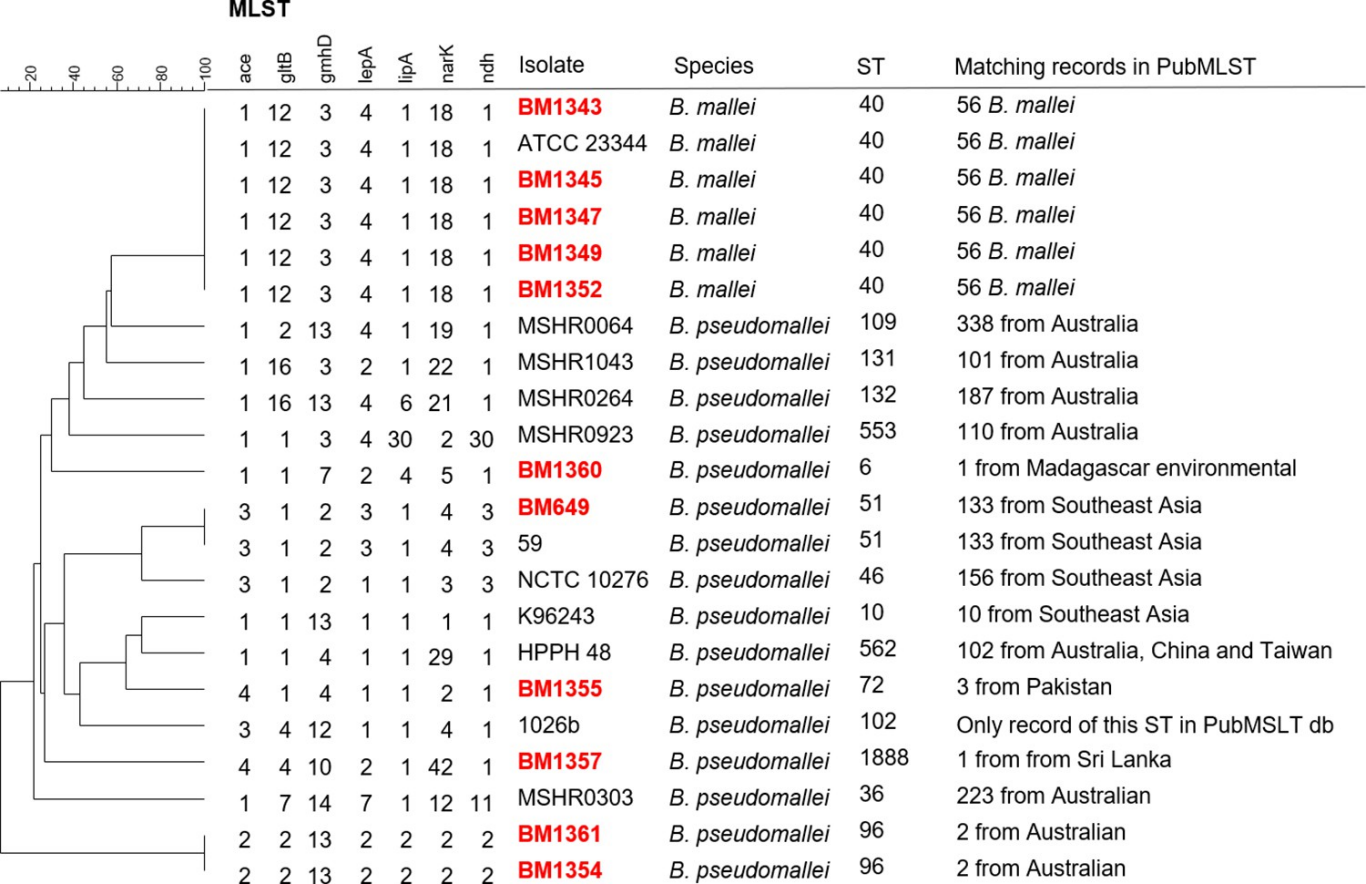
MLST analysis of *Burkholderia* isolates included in this study (red) and 11 additional records retrieved from the PubMLST db based on genetic variation in seven alleles. Sequence types are compared using categorical clustering (values) and clustered with UPGMA using default settings in BioNumerics.

To determine the genetic relationship among the 11 isolates (5 *B*. *mallei* and 6 *B*. *pseudomallei*), a MLST analysis was performed (Fig 1). All *B*. *mallei* isolates were ST-40, indicating the clonality of the species [40]. The *B*. *pseudomallei* isolates were genetically more diverse. BM649 (NCTC 04845) ST-51 is a representative strain for isolates putatively from Southeast Asia, as all 133 records with ST-51 were retrieved from strains derived from this region. BM1357 (06–711) with ST-1888 of which we lack epidemiological data matched with one record from an isolate from Sri Lanka. BM1354 (Holland) and BM1361 (006-00772/2003) were ST-96. Two records with isolates from Australia contained the same ST. BM1360 (PITT 5691) with ST-6 of which we lack epidemiological data matched with one record from an environmental isolate from Madagascar. BM1355 (PITT 521) with ST-72, a human isolate from Pakistan, is one of the three records in the PubMLST db with this ST. All three records with ST-72 are from isolates from Pakistan.

### Antimicrobial susceptibility testing

To be able to correlate the outcome of the proteome analysis to antimicrobial susceptibility, the antimicrobial susceptibility of 11 *B*. *mallei* and *B*. *pseudomallei* isolates to 11 antimicrobials was determined. In Table 3 the minimum inhibitory concentrations of E-tests against amoxicillin/clavulanic acid, ampicillin/sulbactam, ceftazidime, chloramphenicol, ciprofloxacin, doxycycline, imipenem, piperacillin, trimethoprim-sulfamethoxazole, meropenem and tigecycline are listed.

**Table 3:** Minimum inhibitory concentrations (MIC) (μg/mL) of tested antimicrobials against tested *B*. *pseudomallei* and *B*. *mallei* strains.

Strain no.	Species	Amoxicillin/clavulanic acid ≤8/4 S^a^,16/8 I^b^ and ≥32/16 R^c^	Ampicillin/sulbactam ≤8/4 S, 16/8 I and ≥32/16 R	Ceftazidime ≤8 S, 16 I and ≥32 R	Chloramphenicol ≤8 S, 16 I and ≥32 R	Ciprofloxacin ≤1 S, 2 I and ≥4 R	Doxycycline ≤4 S, 8 I and ≥16 R	Imipenem ≤4 S, 8 I and ≥16 R	Piperacillin ≤16 S, 32-64 I and ≥128 R	trimethoprim-sulfamethoxazole ≤2/38 S, ≥4/76 R	Meropenem ≤4 S, 8 I and ≥16 R	Tigecycline ≤4 S, 8 I and ≥16 R
BM1343	*B*. *mallei*	0.75	1.5	0.75	0.5	0.23	0.064	0.125	1	0.23	0.125	0.125
BM1345	*B*. *mallei*	0.75	3	4	>256	8	2	0.25	3	>32	0.125	4
BM1347	*B*. *mallei*	0.5	1.5	0.75	1	0.25	0.047	0.125	4	<0.002	0.064	0.125
BM1349	*B*. *mallei*	1	1.5	1.5	12	1.5	0.32	0.125	1.5	<0.002	0.064	0.19
BM1352	*B*. *mallei*	3	4	2	3	0.25	0.064	0.19	3	0.016	0.125	0.094
BM1354	*B*. *pseudomallei*	3	4	0.75	8	1.5	3	0.5	0.5	1.5	0.5	16
BM1355	*B*. *pseudomallei*	1	2	0.5	1	3	0.25	0.38	1.5	2	0.5	1.5
BM1357	*B*. *pseudomallei*	3	4	1	8	3	1.5	0.25	2	0.25	0.38	6
BM1360	*B*. *pseudomallei*	1	1.5	0.5	16	4	2	0.19	1	1	0.38	4
BM1361	*B*. *pseudomallei*	12	3	0.75	48	6	4	1	1.5	1	0.5	32
BM649	*B*. *pseudomallei*	4	8	1.5	12	8	1.5	0.38	2	1.5	0.75	24

^a^ S) susceptible; indicates there is a high probability of a favorable treatment outcome when treated with this antimicrobial(s)

^b^(I) Intermediate susceptible; indicates a uncertain outcome when treated with this antimicrobial(s)

^c^(R) Resistant, which indicates there is a low probability of a favorable treatment outcome when treated with this antimicrobial(s) (45).

The standard treatment of melioidosis is first intravenous ceftazidime or meropenem followed by oral trimethoprim-sulfamethoxazole or doxycycline or both for a longer time period [41]. Therefore, most studies investigating the spread of antimicrobial resistance in *B*. *mallei* and *B*. *pseudomallei* isolates are conducted using these antimicrobials. All isolates is our study are susceptible for ceftazidime, imipenem and meropenem, which is in line with previous results [25, 42–44]. Furthermore, all isolates were susceptible for piperacillin, doxycycline and only one isolate was intermediate susceptible for amoxicillin/clavulanic acid and ampicillin/sulbactam which is in line with previous studies, as well [25, 42–44]. Next, approximately 10% of the *B*. *mallei* and *B*. *pseudomallei* isolates are resistant for trimethoprim-sulfamethoxazole, which is also reflected by one trimethoprim-sulfamethoxazole resistant *B*. *mallei* (BM1345) out of 11 isolates in our study [25, 43]. Antimicrobial susceptibility was more often decreased for ciprofloxacin, tigecycline and chloramphenicol in *B*. *pseudomallei* compared to a previous study [25].

### Generating MS-spectra and identification of isolates

Between 11,915 to 13,359 MS scans and 32,673 to 45,294 MS/MS scans were obtained from the 11 isolates tested (Table 4). The identification of *B*. *pseudomallei* isolates was based on 173 to 432 discriminative peptides using the proteome2pathogen application. *B*. *mallei* isolates were identified based on 68 to 96 discriminative peptides (Table 5).

**Table 4:** On a Orbitrap Fusion Lumos mass spectrometer generated MS-spectra (MS scans and MS/MS scans) of each isolate included and the number of identified peptides using the MS2peptides-DB.

Micro-organism	Sample nr.	# of MS Scans	# of MS/MS Scans	Peptide-Spectrum Matches	Number of unique peptide sequences
*B*. *mallei*	BM1343	13307	44365	10692	6913
*B*. *mallei*	BM1345	13359	45294	11124	7110
*B*. *mallei*	BM1347	12529	41094	8577	5743
*B*. *mallei*	BM1349	12911	43505	9527	6109
*B*. *mallei*	BM1352	12716	40634	9858	6358
*B*. *pseudomallei*	BM1354	12461	41342	8068	5523
*B*. *pseudomallei*	BM1355	12353	40168	8326	5429
*B*. *pseudomallei*	BM1357	12159	32673	6538	3849
*B*. *pseudomallei*	BM1360	11915	41423	8998	5917
*B*. *pseudomallei*	BM1361	12538	41708	8910	5891
*B*. *pseudomallei*	BM649	12903	44832	9534	6554

**Table 5:** MS-spectra analyzed using PEAKS 7.5 in combination with MS2peptides-DB and http://proteome2pathogen.com/.

Species	Sample nr.	Number of unique peptide sequences after I to L replacement	Highest nr. of discriminative peptides on species level	Total nr. of peptides identified from the identified species	Identified microorganism	Second best result on (first false positive)^a^	Second species identification (first false positive)
*BM* ^*b*^	BM1343	6715	96	6559	*BM*	5	*B*. *gladioli*
*BM*	BM1345	6931	92	6730	*BM*	17	*BP*
*BM*	BM1347	5578	74	5405	*BM*	3	*B*. *oklahomensis*
*BM*	BM1349	5923	82	5764	*BM*	5	*B*. *gladioli*
*BM*	BM1352	6150	68	5958	*BM*	7	*BP*
*BP* ^*c*^	BM1354	5355	259	5233	*BP*	6	*B*. *gladioli*
*BP*	BM1355	5266	331	5098	*BP*	2	*B*. *gladioli*
*BP*	BM1357	3774	173	3621	*BP*	7	*BM*
*BP*	BM1360	5792	268	5583	*BP*	5	*B*. *oklahomensis*
*BP*	BM1361	5795	257	5641	*BP*	7	*B*. *gladioli*
*BP*	BM649	6388	432	6223	*BP*	4	*B*. *cepacia*

^a^ Second highest number of discriminative peptides on species level

^b^ BM: *B*. *mallei*

^c^ BP: *B*. *pseudomallei*.

### Maximizing peptide identifications using a custom database

To maximize the number of identified peptides from the proteome of *B*. *mallei* and *B*. *pseudomallei* isolates, MS-data was analyzed using the specific BPC-DB that contained all annotated proteins of *B*. *mallei* and *B*. *pseudomallei* using the SwissProt database on 14-september 2018, Table 6. Of the peptide-matched MS-spectra a portion of the peptides was identified multiple times. In the eleven samples, between 3,820 and 7,653 (mean 6,385) unique peptides were identified per sample. Some peptides are indistinguishable from other MS-spectra because the only differences in the amino acid sequence of these peptides relate to iso-leucine and leucine substitutions. In the studied samples between 75 and 222 of these indistinguishable peptides were identified.

**Table 6:** Overview of the results of the number of identified peptides using the BPC-DB.

Injected micro- organism	Sample nr.	Peptide-Spectrum Matches	Number of unique peptide sequences	Number of unique peptide Sequence after I to L replacement
*B*. *mallei*	BM1343	11625	7653	7431
*B*. *mallei*	BM1345	11298	7337	7161
*B*. *mallei*	BM1347	9128	6206	6035
*B*. *mallei*	BM1349	10263	6691	6483
*B*. *mallei*	BM1352	10160	6622	6404
*B*. *pseudomallei*	BM1354	8591	5940	5766
*B*. *pseudomallei*	BM1355	9168	6074	5901
*B*. *pseudomallei*	BM1357	6476	3820	3745
*B*. *pseudomallei*	BM1360	9217	6110	5976
*B*. *pseudomallei*	BM1361	9810	6608	6497
*B*. *pseudomallei*	BM649	10308	7170	6994

### Analysis of expressed resistance related proteins against β-lactam and carbapenem antimicrobials

All isolates included in this study were susceptible to β-lactam and carbapenem antimicrobials (Table 3). The maximum number of peptides related to β-lactamase related proteins was 12. In Table 7, the identified peptides from β-lactamases per isolate are described. In all tested isolates peptides were identified derived from PenA-like β-lactamases. Peptides obtained are likely to be derived from a PenA-like β-lactamase but without specific amino acid mutation known in PenA, OXY-2, CTX-M-1 and Toho-1 to confer resistance. Of the identified peptides, two peptides, EPELNTALPGDER and EPELNTALPGDERDTTTPAAMAASVHR, variants are described that contain amino acid mutations conferring antimicrobial resistance [46, 47]. Based on their susceptibility to β-lactam and carbapenem, the PenA proteins identified in our selection of *Burkholderia* species do not contain mutations that confer antimicrobial resistance. Next, six other proteins containing a metallo- β-lactamase domain were demonstrated (Table 7). However, no experimental data in literature could be found indicating that one of these proteins could confer β-lactamase resistance.

**Table 7:** Number of detected peptides from (potential) β lactamases or carbapenemases.

Protein		PenA	Metallo-beta-lactamase superfamily protein	Metallo-beta-lactamase family protein	Metallo-beta-lactamase family protein	Metallo-beta-lactamase family protein	Putative metallo-beta-lactamase family protein	Putative metallo-beta-lactamase family protein
UniProtKB^a^		Q934I1^b^	A0A0F6GHY2^c^	A0A0H2WKH9^c^	A3NJW9^c^	A0A088Y9Y7^c^	Q63J01^c^	Q63UN5^c^
			now A0A069BAE1			now A0A0E1UI06		
Species	BM nr.							
BM^d^	BM1343	10	9	-	-	-	-	-
BM	BM1345	8	7	-	-	-	-	-
BM	BM1347	7	2	4	-	-	-	-
BM	BM1349	7	5	2	-	-	-	-
BM	BM1352	7	8	-	-	-	-	-
BP^e^	BM1354	4	4	-	4	-	-	-
BP	BM1355	3	-	-	-	12	-	-
BP	BM1357	6	-	-	4	-	-	-
BP	BM1360	2	-	-	5	-	-	-
BP	BM1361	5	3	-	5	-	7	-
BP	BM649	4	4	-	8	-	7	2

^a^Protein knowledgebase

^b^PenA family class A extended-spectrum β-lactamase

^c^ UniProt KB of potential metallo-β-lactamase family proteins based on a β-lactamase domain in AA-sequence of the proteins

^d^ BM: *B*. *mallei*

^e^ BP: *B*. *pseudomallei*

### Analysis of expressed proteins potentially involved in co-trimoxazole and tigecycline resistance

In the tested *B*. *pseudomallei* no resistance to the antimicrobial combination drug co-trimoxazole was discovered. Only one of the tested *B*. *mallei* isolates (isolate BM1345) was resistant against co-trimoxazole (trimethoprim-sulfamethoxazole MIC >32 μg/ml).

Trimethoprim and sulfamethoxazole inhibit dihydropteroate synthase (DHPS) and dihydrofolate reductase (DHFR), respectively, which inhibits the tetrahydrofolic acid synthesis pathway [48]. Mutations in DHPS and DHFR could confer resistance to co-trimoxazole. In BM1345 no peptides could be identified that demonstrated the presence of DHPS or DHFR enzymes. Only one peptide of dihydrofolate synthetase (FolC), GTP cyclohydrolase I (FolE) and hydroxymethyl-dihydropterin pyrophosphokinase (FolK), which are three other proteins of the tetrahydrofolic acid synthesis pathway were identified [48]. In the other tested isolates sporadic peptides of the tetrahydrofolic acid synthesis pathway were identified and only one peptide of DHPS was detected, while peptides of DHFR couldn’t be demonstrated.

Next, in the identified proteins based on the database search using BPC-DB, no peptides were detected from tigecycline enzymatic inactivating proteins or ribosomal protection proteins, while three *B*. *pseudomallei* isolates had a tigecycline resistance phenotype [49].

### Analysis of expressed proteins related to efflux pumps in *B*. *mallei* and *B*. *pseudomallei* related to reduced antimicrobial susceptibility

The number of identified peptides and proteins from the three best characterized efflux pumps (AmrAB-OprA, BpeAB-OprB and BpeEF-OprC) involved in antimicrobial resistance against chloramphenicol, ciprofloxacin or trimethoprim-sulfamethoxazole were analyzed [50].

In all *B*. *pseudomallei* and *B*. *mallei* isolates, most peptides detected corresponded to the BpeAB-OprB efflux pump (Table 8). Moreover, expression of BpeA was demonstrated in all isolates by eight or more peptides. Next, in all eleven isolates expression of two out of the three proteins of the BpeAB-OprB efflux pump was demonstrated. In addition, in all tested *B*. *pseudomallei*, two to five peptides of AmrAB-OprA multidrug efflux pump could be detected, while in *B*. *mallei* no peptides were present that were derived from AmrAB-OprA. This corresponds with literature, that at least some, *B*. *mallei* lack AmrAB-OprA expression [47]. Next, efflux pump BpeEF-OprC peptides were only detected in two of the five *B*. *mallei* isolates and not in the tested *B*. *pseudomallei* isolates. Other efflux pump related proteins were detected. However, these were related to efflux of metals (copper, silver, magnesium or cobalt) or only one protein of a putative efflux system was identified (S2 Table).

**Table 8:** Number of detected peptides from efflux pumps.

		AmrAB–OprA			BpeAB–OprB			BpeEF–OprC		
Strain no.	Species	AmrA	AmrB	OprA	BpeA	BpeB	OprB	BpeE	BpeF	OprC
BM1343	BM^a^				16	11	20			
BM1345	BM				18	10		2		3
BM1347	BM				13	6	15			
BM1349	BM				17	8	16	3		5
BM1352	BM				16	11	13			
BM1354	BP^b^			2	12	6	10			
BM1355	BP	5		4	10	8	13			
BM1357	BP		2		9		8			
BM1360	BP	4			14	8	13			
BM1361	BP	3			15	8	18			
BM649	BP	2			8	9	13			

^a^ BM: *B*. *mallei*

^b^ BP: *B*. *pseudomallei*

Expression of an efflux pump does not necessarily indicate reduced susceptibility, but a correlation between expression levels and susceptibly against chloramphenicol, co-trimoxazole, fluoroquinolones, macrolides, sulfamethoxazole, tetracycline and trimethoprim could indicate if an efflux pump influences antimicrobial susceptibility [50]. However, there was no clear correlation found between the expressed proteins and a reduced antimicrobial susceptibility to chloramphenicol, ciprofloxacin and/or trimethoprim-sulfamethoxazole. Moreover, the experimental set-up was to identify as many as possible of the expressed proteins and not to quantitatively determine the amount of protein that is expressed. Therefore, with the obtained data set, it is not possible to determine whether efflux pump proteins are over-expressed. Nevertheless, we think it is likely that based on the high numbers of identified peptides from the efflux pump BpeAB-OprB, it can be assumed that this efflux pump is highly expressed in *B*. *mallei* and *B*. *pseudomallei* at least under standard laboratory conditions.

### Analyzing the expressed virulence related proteins

The developed BPC-Virulence-DB was used to screen which peptides were expressed under standard culture conditions (overnight culture on a blood agar plate). Only the virulence factors of which ≥2 peptides were identified in one of the isolates are reported. Peptides of six different virulence factors were identified (Table 9). In more detail, the coverage and the number of peptides identified of each protein analyzed of the studied virulence related proteins are described (S3 Table). Capsule I and T2SS proteins were identified in all isolates. A high number of peptides derived from proteins related to flagella were identified in *B*. *pseudomallei* isolates. As could be expected there were only a few flagella related proteins identified in non-motile *B*. *mallei* [51]. Expression of type VI secretion system (T6SS-1) proteins was demonstrated in all tested *B*. *pseudomallei* and 2 of the 5 *B*. *mallei*. Moreover, in all *B*. *pseudomallei* studied, expression of clpV (ATP-dependent protease ClpV) and TssM (type VI secretion system membrane subunit TssM) could be demonstrated. Expression of TssC and TssD was demonstrated in 4 out of the 6 *B*. *pseudomallei* isolates. TssC expression was demonstrated in BM1354, BM1357, and BM1360, while TssD expression was demonstrate in BM1354, BM1355, BM1357 and BM1360. In *B*. *mallei* BM1343 expression of T6SS-1 related proteins could be demonstrated, while only TssM expression was demonstrated in one other *B*. *mallei* (BM1352) isolate. Burtnick *et al*. showed that TssM is important in regulating the innate immune response. Expression is coregulated with that of T6SS-1 but not exported by T6SS-1. The secretion is mediated by the T2SS of which in all isolates tested expression of one or more protein was demonstrated [17].

**Table 9:** The number of proteins and peptides (in parentheses) identified for each virulence factor.

BM nr.	Flagella	Capsule I	T6SS-1	T2SS	Surface polysaccharide	Type IV pili
*B*. *mallei*						
BM1343	-	8 (47)	6 (37)	3 (10)	-	-
BM1345	2 (4)	7 (39)	-	3 (9)	-	1 (3)
BM1347	1 (2)	6 (39)	-	3 (6)	-	1 (2)
BM1349	1 (3)	8 (34)	-	2 (6)	-	1 (4)
BM1352	-	9 (37)	1 (2)	3 (9)	-	-
*B*. *pseudomallei*						
BM1354	17 (77)	6 (38)	4 (11)	3 (12)	1 (4)	-
BM1355	15 (75)	8 (41)	3 (8)	2 (11)	1 (4)	-
BM1357	12 (59)	5 (20)	4 (10)	1 (6)	-	-
BM1360	16 (59)	6 (44)	4 (17)	2 (9)	1 (6)	-
BM1361	18 (83)	6 (38)	3 (10)	2 (9)	1 (3)	-
BM649	9 (48)	9 (46)	2 (7)	4 (14)	1 (3)	1 (3)

In all except one *B*. *pseudomallei* isolate, poly-beta-1,6 N-acetyl-D-glucosamine export porin was detected (PgaA), which was not identified in *B*. *mallei*. This surface polysaccharide PgaA is assumed to be involved in biofilm formation [52]. In 4 isolates (3 *B*. *mallei* and 1 *B*. *pseudomallei*) multiple peptides from a protein characterized as virulence factor of type IV pili, were identified.

Type three secretion system (T3SS) proteins were not identified except for one protein BopE (wp.04528812.1) in BM1361 of which two peptides were identified. No proteins involved in BspIR quorum-sensing system were detected.

### Proteins involved in polyketide and non-ribosomal peptide synthetase

Expression of proteins of in Table 2 described PKS/NRPS clusters were determined for all 11 investigated isolates. This additional search for the expressed proteins from known PKS/ NRPS clusters was executed because these were not well represented in the VFDB, while these proteins are often involved in the production of secondary metabolites, which are essential for *B*. *mallei* and *B*. *pseudomallei* pathogenesis [39]. In S4 Table, the coverage and the number of peptides identified of each protein analyzed of the studied PKS/NRPS clusters are described. From the PKS/NRPS clusters investigated, the malleobactin cluster is expressed by all 6 *B*. *pseudomallei* and 5 *B*. *mallei* isolates analyzed. In all these isolates at least, expression of 4 proteins of the malleobactin cluster was demonstrated. The malleilactone cluster was expressed in 4 *B*. *pseudomallei* and 2 *B*. *mallei* isolates based on the expression of at least 4 proteins of this cluster. In the 6 *B*. *pseudomallei* isolates analyzed, also expression of three other PKS/NRPS clusters was indicated by the identification of >2 proteins namely, two putative PKS/NRPS clusters (BPSS0481–BPSS0487 and BPSS1181–BPSS1199) and the bactobolin synthase cluster (BPSS1166–BPSS1174). Expression of 7 of the 9 proteins of the bactobolin synthase clusters was demonstrated in all 6 studied *B*. *pseudomallei* isolates.

Analysis of a total of 15 PKS/NRPS clusters indicated the expression of 12 and 7 clusters in *B*. *pseudomallei* BM649 and BM1355 respectively. Expression of other NRPS/PKS clusters other than the two involved in the production of malleobactin and malleilactone in the 5 *B*. *mallei* couldn’t be demonstrated based on the expression of >2 proteins in a *B*. *mallei* isolate.

## Discussion

The proteomes of different *B*. *mallei* and *B*. *pseudomallei* isolates were investigated to determine if virulence, antimicrobial resistance, PKS/NRPS clusters related proteins are expressed by all or a subset of the *B*. *mallei* and *B*. *pseudomallei* isolates. Results demonstrate the feasibility of in-depth characterization of *B*. *mallei* and *B*. *pseudomallei* isolates by proteome analysis using LC-HRMS/MS.

To characterize the proteome different databases searches were used. Moreover, a database that contains all microbial proteins expressed is overwhelming and complicates database searches to identify the peptides in the sample based on the analyzed MS/MS-spectra and could lead to a lower number of identified peptides of the proteins of interest [53]. To confirm the identity of the used isolates and to determine the number of unique peptides in each analyzed isolate a database (MS2peptides-DB) was previously constructed with known human pathogenic and related species [15, 54]. Together with the web-based application, proteome2pathogen.com, it enables identification of most clinical relevant bacteria [15]. Previously, this application enabled the identification of bacteria grown in blood culture flasks including *B*. *mallei* and *B*. *pseudomallei* isolates based on 7 to 13 and 14 to 53 discriminative peptides, respectively [15]. Next, the proteomes analyzed in this study were derived from blood culture ager plates and analyzed with another LC-MS/MS system, which increased the yield of peptides identified of tested *B*. *mallei* and *B*. *pseudomallei* (Table 5). Thereby, the investigated isolates could be identified based on 68 to 96 and 173 to 432 discriminative peptides in *B*. *mallei* and *B*. *pseudomallei*, respectively. The identification of *B*. *mallei* and *B*. *pseudomallei* based on a high number of discriminating peptides can increase the confidence in the diagnoses of melioidosis and glanders. Moreover, until now, diagnosis of these diseases is often cumbersome because of the diverse clinical manifestations, inadequacy of conventional diagnostic methods and lack of diagnostic facilities [13, 14].

The MS2peptides-DB doesn’t contain the whole protein diversity of *B*. *pseudomallei* and *B*. *mallei*. Therefore, BPC-DB, that contains only the annotated diversity of the proteomes of *B*. *pseudomallei* and *B*. *mallei* was used to analyze expression of investigated isolates. By using the BPC-DB the number of identified peptides increased. The increase in identified peptides is limited to maximal 10%, indicating that for general profiling and identification of *B*. *pseudomallei* and *B*. *mallei* the MS2peptides database is sufficient and adding additional proteomes of additional *B*. *pseudomallei* and *B*. *mallei* isolates has a limited added value.

For effective treatment of infections with *B*. *pseudomallei* and *B*. *mallei* it is important to determine antimicrobial susceptibility of the isolated bacterium swiftly. Therefore, one of the objectives was to determine how and what type of information regarding antimicrobial resistance can be deduced from the measured proteomes. Results showed that tested *B*. *pseudomallei* and *B*. *mallei* were susceptible to all tested β-lactam and carbapenem antimicrobials. In accordance with resistance-testing on agar plates in the measured proteomes no peptides were identified linked to ESBLs or carbapenemases. Reduced antimicrobial susceptibility towards other types of antimicrobials were not detected by the method used. With specific database searches, no peptides were identified related to cotrimoxazole resistance in a cotrimoxazole resistant *B*. *mallei* [48]. Also, no peptides were detected related to tigecycline / tetracycline inactivating enzymes or ribosomal protection proteins in *B*. *pseudomallei* with reduced susceptibly for tigecycline [32].

Overexpression of efflux pumps in the cell wall of bacteria can extrude antimicrobials out of the cell, which makes the bacteria less susceptible for multiple antimicrobials, like tetracycline, tigecycline, chloramphenicol, trimethoprim, fluoroquinolones, sulfamethoxazole, aminoglycosides and macrolides [50]. Therefore, the expression of efflux pumps was studied. From the number of identified peptides from the identified efflux pump proteins from the three best characterized efflux pumps involved in antimicrobial resistance, there was no clear correlation to reduced antimicrobial resistance detected. Most peptides were identified from the BpeAB-OprB efflux pump, which could indicate that this efflux pump is constitutively expressed by *B*. *pseudomallei* and *B*. *mallei*, at least under standard laboratory conditions.

Antimicrobial resistance can be induced when the agent is in contact with the antimicrobial agent [55, 56]. Samples studied here are from bacteria cultured without antimicrobial pressure and induced antimicrobial resistance proteins therefore might not be expressed and detected. Furthermore, there are numerous potential causes of antimicrobial resistance in which multiple genes, mutations and expression levels of the proteins could be involved [47]. Our searches in the proteomes and whole genome sequences are executed on antimicrobial resistance mechanism described in literature [46–50, 57, 58] but could not elucidate the causes of phenotypically demonstrated antimicrobial resistance. Previously, Rhodes and Schweizer noted that our overall understanding of resistance in *B*. *pseudomallei* and *B*. *mallei* is still quite rudimentary [47]. Possibly, therefore it is possible that the causes of antimicrobial resistance weren’t recognized in our study. Another cause could be that antimicrobial resistance proteins has to be to be induced in *B*. *pseudomallei* and *B*. *mallei*, or is multifactorial which wasn’t investigated in this study. Another point of interest are the virulence factors that are expressed by the isolated *B*. *mallei* and *B*. *pseudomallei*. Using a custom database (BPC-Virulence-DB) based on the virulence factors described in the VFDB (Virulence Factors DataBase), six different virulence factors were detected in the tested *B*. *mallei* and *B*. *pseudomallei* isolates [33]. All isolates expressed capsule I and type II secretion system (T2SS) proteins. All *B*. *pseudomallei* expressed a high number of flagella related proteins whereas only a limited number of flagella related proteins were expressed by *B*. *mallei*, what corresponds to the expectation that *B*. *mallei* in contrast to *B*. *pseudomallei*, being non-motile [59]. Furthermore, expression of Type VI secretion system (T6SS) proteins, a surface polysaccharide and type IV pili proteins was demonstrated.

Finally, the expression of PKS/NRPS clusters was studied. These often produce secondary metabolites that are essential for *B*. *mallei* and *B*. *pseudomallei*. The proteome and the secondary metabolism of bacteria are influenced by its environment [39]. Expression of proteins involved in production of certain secondary metabolites could be an indication that the associated metabolites are produced. In all isolates a high number of peptides was detected from the malleobactin gene cluster, which indicates the expression of the genes involved in malleobactin E synthesis. Bacteria secrete siderophores, such as malleobactin E to scavenge iron, which is essential during infection for bacterial survival in the human body.

Proteins of the malleilactone cluster are expressed in multiple isolates (in least 2 *B*. *mallei* and 3 *B*. *pseudomallei* based on >2 proteins expressed), which indicates that some isolates express malleilactone under standard laboratory culture conditions, while others do not. This indicates that expression of malleilactone is, next to environmental conditions, also determined by the isolate investigated [35, 39]. Malleilactone is cytotoxic to several bacteria, non-mammalian models and mammalian cells [35, 39]. Proteins of the bactobolin synthase cluster were highly expressed by all 6 *B*. *pseudomallei* isolates indicating the continued expression of this cytotoxic compound by *B*. *pseudomallei* under standard laboratorial culture conditions, while in only one of the 5 examined *B*. *mallei* bactabolin was expressed (expression of 2 proteins was demonstrated). Bactobolins are also reported to be cytotoxic to several bacteria, non-mammalian models and mammalian cells [60].

The protein diversity in *B*. *mallei* seems limited, which is in line with expectations because *B*. *mallei* isolates are genetically highly related. The examined *B*. *pseudomallei* proteome profiles are more diverse. BM649 expressed most proteins of PKS/NRPS clusters analyzed, indicating that multiple secondary metabolites are expressed simultaneously. Because BM649 (ATCC 15682) was isolated in 1935 and likely has undergone many cultivation passages, extrapolation of the expressed proteome of BM649 to an infection with a wildtype of *B*. *pseudomallei* is not recommended [61]. However, the presence of these PKS/NRPS clusters overtime and expression of the proteins in these clusters makes this an interesting strain to study the expression of secondary metabolites by *B*. *pseudomallei*.

Next, the diversity in the expressed proteins per isolate indicates that strains can exhibit distinct phenotypes. Proteins related to virulence including PKS/NRPS clusters that encode for secondary metabolites are demonstrated.

For several virulence factors (as T2SS, T3SS, T6SS-1, flagella, capsule I, surface polysaccharide, BspIR quorum-sensing system and type IV pili) an effect on virulence of *B*. *pseudomallei* or *B*. *mallei* is demonstrated [2, 17, 34, 35, 39, 62]. In this study we demonstrate the expression of proteins cultured under standard laboratory conditions, as described in material and methods, in the analyzed isolates. Proteins of capsule I, T2SS, efflux pump BpeAB-OprB and malleobactin synthase cluster (‘pan-expressed-proteins’) are expressed, while proteins of flagella, T6SS-1, surface polysaccharide, bactobolin synthase cluster and a putative antibiotic cluster (BPSS1181-BPSS1196) are expressed by the tested *B*. *pseudomallei* but not or nearly not by the tested *B*. *mallei*. Expression of IV pili, efflux pumps AmrAB-OprA and BpeEF-OprC and other PKS/NRPS related proteins are expressed by only a subset of the tested isolates.

The pathogenesis of *B*. *pseudomallei* and *B*. *mallei* is multifactorial process, which is regulated by environmental factors which the bacterium can anticipate [62]. For example T6SS-1, ferric uptake regulators and siderophore production are upregulated when availability of iron is limited, which is often the case during infection [62, 63]. Therefore, it is intriguing that under standard laboratory conditions in which iron isn’t restricted, malleobactin (a siderophore) is expressed in all *B*. *mallei* and *B*. *pseudomallei* isolates and T6SS-1 in *B*. *pseudomallei* and in two of the five *B*. *mallei* investigated.

Next, to proteins or protein complexes also a role in virulence of secondary metabolites is proposed in the pathogenicity of *B*. *mallei* and *B*. *pseudomallei* [39]. Despite *B*. *pseudomallei* poses a health risk and is endemic in some regions of the world both species (*B*. *pseudomallei* and *B*. *mallei*) are designated Tier 1 Select agents because they both can be deliberately misused with significant potential for mass casualties. In this study we demonstrated the expression of pan-expressed-proteins in all 11 isolates. Assuming that these pan-expressed proteins are also expressed during infection makes these pan-expressed-proteins are highly interesting targets for drug development.

This study demonstrated that proteome analysis can be used to extract important expressed characteristics of *B*. *mallei* and *B*. *pseudomallei*. However, the presented results do not claim that the expression of all important properties of *B*. *mallei* and *B*. *pseudomallei* were identified. There are likely other properties which are also important for *B*. *mallei* and *B*. *pseudomallei* to be successful as a pathogen, including expression of proteins not observed in this study.

The complexity of *B*. *mallei* and *B*. *pseudomallei* in general, the potential variation of protein expression under different conditions, and the gaps in knowledge concerning the proteins involved in antimicrobial resistance and possibly virulence as yet complicates the possibility to characterize *B*. *mallei* and *B*. *pseudomallei* in one LC-MS/MS analysis.

In conclusion, the approach presented can be used to identify *B*. *mallei* and *B*. *pseudomallei* reliably but also to characterize the presence of factors that putatively contribute to the pathogenesis of *B*. *mallei* and *B*. *pseudomallei*.

## Supporting information

S1 Table Search parameters for peptide identification. (XLSX)

S2 Table Additionally expressed putative efflux related proteins. (XLSX)

S3 Table Proteins identified of known virulence factors. (XLSX)

S4 Table Proteins identified for each known NRPS or PKS cluster. (XLSX)
